# Surgical Management of Malignant Glioma in the Elderly

**DOI:** 10.3389/fonc.2022.900382

**Published:** 2022-05-26

**Authors:** Julia Klingenschmid, Aleksandrs Krigers, Johannes Kerschbaumer, Claudius Thomé, Daniel Pinggera, Christian F. Freyschlag

**Affiliations:** Department of Neurosurgery, Medical University Innsbruck, Innsbruck, Austria

**Keywords:** malignant glioma, glioblastoma, elderly, surgery, survival

## Abstract

**Background:**

The median age for diagnosis of glioblastoma is 64 years and the incidence rises with increasing age to a peak at 75-84 years. As the total number of high-grade glioma patients is expected to increase with an aging population, neuro-oncological surgery faces new treatment challenges, especially regarding aggressiveness of the surgical approach and extent of resection. In the elderly, aspects like frailty and functional recovery time have to be taken into account before performing surgery.

**Material & Methods:**

Patients undergoing surgery for malignant glioma (WHO grade III and IV) at our institution between 2015 and 2020 were compiled in a centralized tumor database and analyzed retrospectively. Karnofsky Performance Scale (KPS) and Clinical Frailty Scale (CFS) were used to determine functional performance pre- and postoperatively. Overall survival (OS) was compared between age groups of 65-69 years, 70-74 years, 75-79 years, 80-84 years and >85 years in view of extent of resection (EOR). Furthermore, we performed a literature evaluation focusing on surgical treatment of newly diagnosed malignant glioma in the elderly.

**Results:**

We analyzed 121 patients aged 65 years and above (range 65 to 88, mean 74 years). Mean overall survival (OS) was 10.35 months (SD = 11.38). Of all patients, only a minority (22.3%) received tumor biopsy instead of gross total resection (GTR, 61.2%) or subtotal resection (STR, 16.5%). Postoperatively, 52.9% of patients were treated according to the Stupp protocol. OS differed significantly between extent of resection (EOR) groups (4.0 months after biopsy vs. 8.3 after STR vs. 13.8 after GTR, p < 0.05 and p < 0.001 correspondingly). No significant difference was observed regarding EOR across different age groups.

**Conclusion:**

GTR should be the treatment of choice also in elderly patients with malignant glioma as functional outcome and survival after surgery are remarkably better compared to less aggressive treatment. Elderly patients who received GTR of high-grade gliomas survived significantly longer compared to patients who underwent biopsy and STR. Age seems to have little influence on overall survival in selected surgically extensive treated patients, but high preoperative functional performance is mandatory.

## Introduction

Glioblastoma has an incidence of 3.2 cases per 100.000 adults and therefore constitutes the most common malignant primary brain tumor. Median age at diagnosis is 64 years with an increasing incidence with rising age, peaking at 75-84 years (1). Median survival lies between 12-15 months in all patients despite aggressive treatment, being markedly decreased in elderly patients with only 4-5 months from diagnosis (2, 3). As the average age of the population rises, elderly patients represent already up to 25% of all WHO° IV brain tumor patients (4, 5). Thus, treatment options and prognostic factors must be re-evaluated in the face of an aging patient group.

Age per se is known to be a negative prognostic factor in patients with malignant glioma with a statistically significant decrease of survival per each additional year of age (6–9). Further, molecular diagnosis in the older population prominently reveals primary glioblastoma, lacking IDH mutation (10). MGMT promoter methylation can be found in approximately 40-60% of elderly glioblastoma patients, being a favorable prognostic factor in all age groups (11–14).

Performance status has gained more and more impact in the individual assessment of elderly patients regarding their prognosis and eligibility for treatment. Physical wellbeing including organ function and associated comorbidities play a more important role than chronological age alone (15). KPS and more modern score systems assessing frailty help to depict a holistic image of elderly patients including strength, endurance and physiologic function resulting from diseases or diverse medical conditions (16).

Surgery in malignant gliomas aims to prolong overall survival (OS) and progression free survival (PFS), helps to gain histopathological and molecular information as well as, due to the reduction of mass effect, decreases the use of steroids . Yet, for a long time, extensive resection was withheld in the elderly fearing a worse outcome. Recent data, however, underlines the importance and safety of aggressive surgical treatment even in the elderly (17–21).

Following surgery, further oncological treatment in the elderly depends mainly on the overall functional status as benefits of any therapy become more closely balanced with risks of toxicity. Elderly patients with poor performance status often better tolerate single-modality therapy that is radiotherapy or temozolomide alone. Both sole hypofractionated radiotherapy and temozolomide chemotherapy are administered provides good results in elderly patients with poor performance status (11, 22). Recent data, however, favors a combined radiotherapy as well, especially in MGMT-methylated patients, the method of radiation still matter of debate (23).

The WHO defines ‘elderly’ above 65 years of age, therefore data on surgical treatment of malignant glioma in the large cohort of the elderly is started at this age, mostly without further subdivision. Thus, we aimed to analyze the influence of extent of surgical resection on survival in different age groups above 65 years. Furthermore, a literature review was performed with focus on the surgical treatment modalities and compared to our data.

## Material and Methods

A total of 121 patients aged 65 years and above with histologically confirmed WHO grade III and IV tumors who underwent surgical treatment at our institution between 2015 and 2020 were analyzed. Surgical therapy included biopsy (either stereotactic or frameless), subtotal resection (STR) or gross total resection (GTR, defined as EOR > 98% of all contrast-enhancing tumor, as gauged by MRI). STR was defined as partial tumor removal with an EOR >80% in the light of preserving neurological status but with residual nodular enhancement in MRI (24).

Clinical performance was assessed using the Rockwood Clinical Frailty Scale (CFS) and Karnofsky Performance Scale (KPS). Examinations were performed preoperatively, postoperatively and three to six months after surgery. CFS was assessed retrospectively blinded to the outcome data using the functional description and standardized neurological status of the patients, which were documented in patients’ charts. Karnofsky Performance Status Scale (KPS) was prospectively assessed in all patients preoperatively and 3 to 6 months after surgery as an institutional clinical routine.

Neuropathological grading was based on the revised 4th WHO classification of CNS tumors. Presence of IDH1 mutation, as well as nuclear ATRX expression was proven by immunohistochemistry. DNA sequencing was applied to evaluate MGMT promotor methylation, using a cut-off at 8%.

Statistical analysis was performed using IBM SPSS Statistics (IBM SPSS Statistics for Windows, Version 27.0. Armonk, NY. IBM Corp.). Normal distribution of scale data was checked using Kolmogorov-Smirnov test and if normal distribution was not confirmed, Mann-Whitney-U test for unpaired or Wilcoxon and Friedmann test for paired ranked or scale parameters were applied. Spearman’s test was used to assess correlations of non-parametric data. Overall survival was estimated using Kaplan-Meier processing and log-rank tests. Results with p < 0.05 were considered as statistically significant.

## Results

We included 121 patients with an age of 65 years and older in this investigation – 46 females and 75 males. To be precise, 27 patients (22.3%) had an age of 65 – 69 years at time of surgery, 35 patients (28.9%) were 70 – 74 years old, 41 (33.9%) were 75 – 79, 12 (9.9%) were 80 – 84 and 6 (5.0%) were 85 years old or older. Mean age at surgery was 74 years (SD = 5). Mean estimated overall survival (OS) was 10.35 months (CI 95%: 8.26-12.45).

All except to four patients (WHO grade III) showed WHO grade IV tumors. Of all patients, only three (2.5%) showed IDH1 mutation, whereas 111 patients (91.7%) had an IDH1 wildtype tumor. In seven patients (5.8%) IDH1 mutation status was not available due to missing histopathological data.

MGMT promotor methylation was present in 58 patients (47.9%) in contrast to 52 patients (43.0%) where no methylation was found. In ten patients, methylation status was not available. Nuclear ATRX was found to be expressed in specimens of 105 patients (86.8%), not expressed in two (1.7%) and not tested for in 14 (11.6%) patients.

As far as the extent of resection (EOR) is concerned, 27 patients (22.3%) received a biopsy only while 74 patients (61.2%) were treated with gross total resection (GTR). Twenty patients (16.5%) had a subtotal resection (STR). **Table 1** shows the distribution of age groups amongst the different extents of resection.

**Table 1:** EOR according to different age groups.

			65-69	70-74	75-79	80-84	≥ 85
EOR	Biopsy	quantity	9	3	10	2	3
		% of age groups	33.3%	8.6%	24.4%	16.7%	50.0%
		% of total	7.4%	2.5%	8.3%	1.7%	2.5%
	GTR	quantity	18	27	19	7	3
		% of age groups	66.7%	77.1%	46.3%	58.3%	50.0%
		% of total	14.9%	22.3%	15.7%	5.8%	2.5%
	STR	quantity	0	5	12	3	0
		% of age groups	0.0%	14.3%	29.3%	25.0%	0.0%
		% of total	0.0%	4.1%	9.9%	2.5%	0.0%
total		quantity	27	35	41	12	6
		% of total	22.3%	28.9%	33.9%	9.9%	5.0%

A total of 65 patients (53.7%) were treated with a 6-week period of radiotherapy with a radiation dose of 60 Gy and concomitant temozolomide (18). Additionally, fifty patients (41.3%) received adjuvant temozolomide with a mean of 2.2 cycles (SD = 3.72). By default, radiotherapy was performed using a regime of 60 Gy over 6 weeks and temozolomide was administered according to the Stupp protocol in a weight-based manner. Only both in five patients the radiation was adapted to a dose between 30 and 50 Gy, and temozolomide was administered in a low-dose scheme. Sole radiation monotherapy was applied to 14 patients (11.6%). In 42 patients (34.7%) no further treatment was carried out.

Results regarding patient assessment for functional status using KPS and CFS are shown in **Table 2**. KPS stayed stable with a light increase at follow up, whereas CFS remained stable. Changes were not statistically significant (p – ns.)

**Table 2:** Median pre- and postoperative as well as follow-up values for KPS and CFS, including IqR, are depicted.

	preoperatively	postoperatively	3-6 months follow-up
KPS (median (SD))	80 (20)	80 (20)	90 (20)
CFS (median (SD))	3 (1)	–	3 (2)

**Figure 1:**
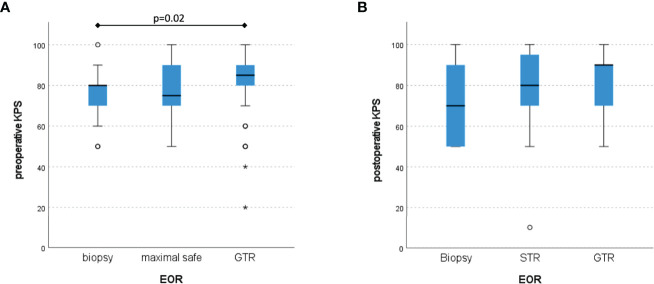
Distribution of KPS according to the different EOR with a significant preoperative difference **(A)**, but non-significant values postoperatively **(B)** (Box plot diagram).

Preoperative KPS and CFS were significant better in the GTR group compared to biopsy and STR (KPS: p < 0.01 and CFS: p < 0.05, respectively). At the follow-up visit after 3 to 6 months, no significant difference in KPS could be shown (p – n.s.), see **Figure 1**.

**Figure 2:**
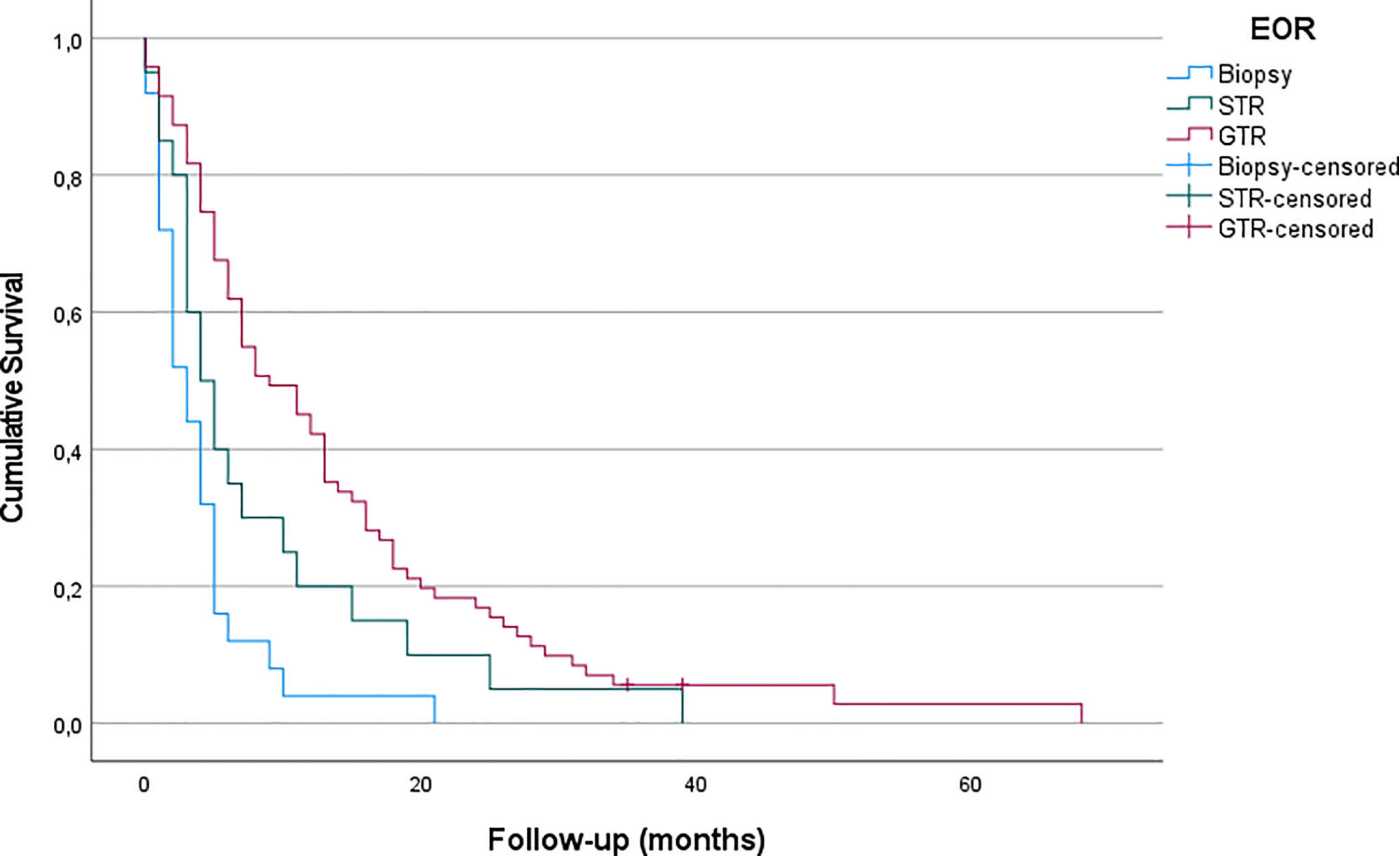
Differences of OS in the treatment groups (biopsy, GTR, STR) are shown in Kaplan-Meier processing. LogRank test Biopsy-GTR: p<0.001.

Patients receiving biopsy had a mean OS of 3.96 months (CI95% = 2.23 – 5.67). After STR, patients lived for a mean of 8.30 months (CI95% = 4.05 – 12.55), while mean OS following GTR was 13.80 months (CI95% = 10.46 – 17.15). **Figure 2** shows the corresponding Kaplan-Meier curves. When examining the significance more closely, looking at EOR in pairs, biopsy versus STR showed no significant difference in OS, while biopsy versus GTR demonstrated a significant difference (p < 0.05), as well as STR versus GTR (p < 0.001).

**Figure 3:**
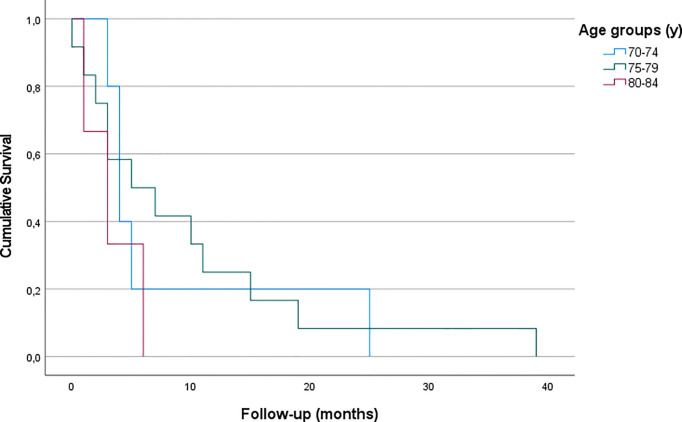
Kaplan-Meier curves for patients of all age groups who received GTR (p - ns).

**Figure 4:**
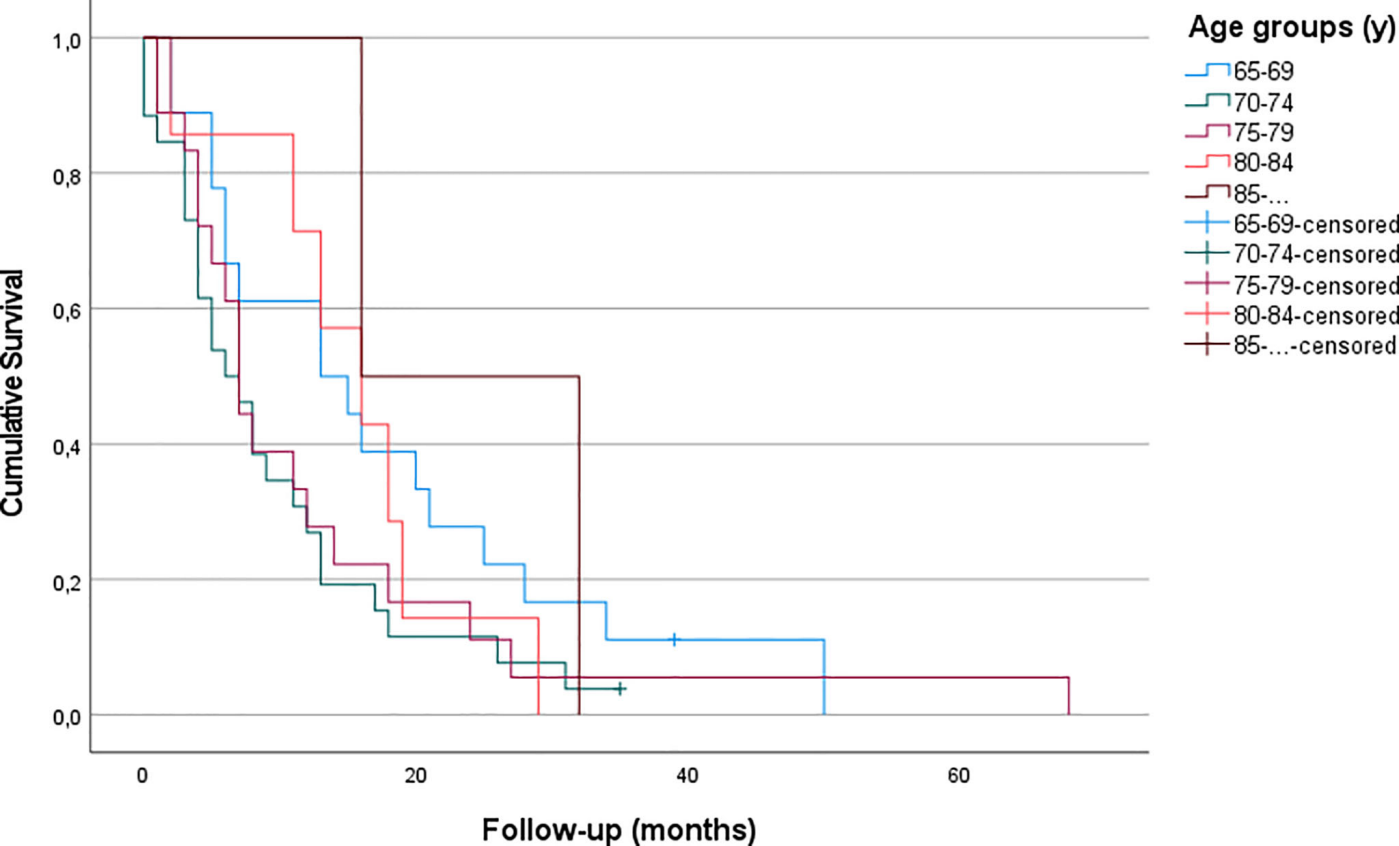
Kaplan-Meier curves for patients of all age groups who were treated with STR (no patients under 70 and over 84 years received STR in our cohort) (p - ns).

Patients who received GTR showed no significant differences in OS with regard to their age (p – n.s.). Furthermore, OS following STR did not differ significantly either (p – n.s.) (**Figures 3**, **4** and **Table 3**).

**Table 3:** Mean and median OS in different age groups receiving GTR and STR.

Age groups	GTR			STR		
	median OS (months)	mean OS (months)	CI 95%	median OS (months)	mean OS (months)	CI 95%
65-69	14.00	17.67	10.76 – 24.57			
70-74	5.00	9.46	5.95 – 12.97	4.00	8.20	.00 – 16.46
75-79	7.00	12.61	5.39 – 19.84	6.00	9.58	3.38 – 15.79
80-84	12.00	15.43	9.31 – 21.55	3.00	3.33	.49 – 6.18
≥ 85.	24.00	24.00	8.32 – 39.68		-	.
Total	7.00	13.80	10.46 – 17.15	9.00	8.30	4.05 – 12.55

No significant differences were present.

## Discussion

Extensive resection benefits overall survival within all elderly age groups, even in the very old. OS after sole biopsy was shorter (approximately 4 months) than after STR (8 months) and GTR (14 months) for elderly patients. Additionally, our findings suggest that patients with good preoperative functional status, as assessed in KPS, are more likely to be treated by extensive surgery.

### Extent of Resection

With most of the elderly patients (61.2%) treated with GTR and more than 50% receiving postoperative therapy according to the Stupp protocol, we aim for an extensive tumor therapy also in this age group. Nearly all our patients` histopathological and molecular testing showed WHO grade IV tumors without IDH1 mutation which matches literature data (19, 25, 26).

Our results are congruent to previous findings, indicating that a more aggressive surgical approach leads to longer survival (18–20). A retrospective case-control analysis conducted by Chaichana et al. found overall survival (OS) time to be increased by 40% (which equaled 2 months in their cohort) in elderly patients who underwent surgical resection compared to those undergoing needle biopsy. At the same time, surgery-related morbidity was demonstrated to be similar in case of aggressive resection and biopsy (18). This was confirmed in another retrospective study which assessed 178 patients with a median age of 71 years, showing a 2-year-OS three times higher, when the contrast-enhancing tumor was resected completely compared to patients with biopsy alone (19). A systematic review and meta-analysis including more than 12.000 elderly patients confirmed that maximal resections are safe and are associated with longer survival (increased by an average of 7 months in gross total resection compared to biopsy), improved functional recovery and delayed tumor progression while showing no higher rates of mortality or morbidity according to the extent of resection (20). Data of the SEER (Surveillance, Epidemiology, and End Results) cancer registry also found GTR to be associated with improved overall survival (27). Analysis of 20.705 patients harboring glioblastoma found a strong association between EOR and OS, regardless of age. Yet, their OS is lower than our findings, possibly due to historic data. Contrary to our findings, Babu et al. demonstrated a decreased survival in patients aged above 75 years in their series, yet, the other results are in line with our data (EOR, KPS) (28). Niare et al. presented a series of selected patients 80 years or older, which revealed that radical resection of GBM was associated with acceptable survival in contrast to sole biopsy. Moreover, their data underlined the need for adjuvant treatment with the complete Stupp protocol (29, 30). Nevertheless, direct comparison is cumbersome, as the distribution of EOR in their age comparison is not mentioned. A recent review reports data showing GTR to be more effective than STR in achieving longer survival in elderly patients with high-grade glioma as it can significantly improve OS and 3-, 6-, 9-month, and 1-year mortality (21).

Overall, recent literature favors extensive surgical resection also in the elderly, even though uncertainties due to comorbidities and tumor localization remain (19, 20, 25, 26, 28)

### Performance

Geriatric glioblastoma patients with increased frailty have shown to have a higher probability for poor survival with increasing patient age (26). Thus, preoperative functional status should be considered in individual treatment decision making as a more relevant factor than chronologic age. Both KPS and CFS show congruent results at the post-operative follow up in our series and similar to preoperative assessment supporting the importance of proper patient selection. Recent data analyzing 110 elderly patients described an association between preoperatively increased frailty and decreased survival following surgical treatment of geriatric glioblastoma patients. Moreover, an increased comorbidity burden and subtotal resection was associated with poor survival (26). Although our series did not include comorbidities, latter results are in line with our surgical series. Zorman et al. recently proposed both the Elderly Glioblastoma Surgical Score (EGSS) and the Elderly Glioblastoma Oncological Score (EGOS). Both were proven to be capable to estimate the survival of elderly glioblastoma patients, considering age, WHO performance status, surgical intervention and chemoradiotherapy (23).

Limitations of this study are its retrospective character and the potential interrater variability in assessment of the functional scores. Like with most comparable studies, there is a risk of selection bias. Patients with initially higher KPS tend to be treated more aggressively, reflected by the lower KPS in the biopsy cohort also in our study. Additionally, in more eloquent lesions only STR may be possible and outcome with earlier neurologic decline with tumor progression may be inferior. Nevertheless, our data demonstrate a clear survival benefit with aggressive surgery.

## Conclusion

Elderly patients who received GTR of high-grade gliomas live significantly longer compared to patients who underwent biopsy or STR. Age per se seems to have no influence on overall survival in selected extensive operated patients, but good preoperative performance status is mandatory. Thus, we should strive for maximal tumor resection in patients of all ages with malignant glioma. Nonetheless, the process of decision making in patients with high grade brain tumors remains a complex, interdisciplinary process and must imply the individual patient`s expectations and needs.

## Data Availability Statement

The original contributions presented in the study are included in the article/supplementary material. Further inquiries can be directed to the corresponding author.

## Ethics Statement

The studies involving human participants were reviewed and approved by the local ethics committee of the Medical University Innsbruck (Protocol number: AN 1333/2021). The patients/participants provided their written informed consent to participate in this study.

## Author Contributions

JuK: acquisition, analysis of data, interpretation of data and drafting the article. JuK: acquisition and interpretation of data. AK and DP: acquisition, analysis of data and drafting the article. CF and CT: design of the study and revisions. CF: conception/design of the study and interpretation of data. All authors have approved the submitted version and have agreed both to be personally accountable for the author’s own contributions and to ensure that questions related to the accuracy or integrity of any part of the work, even ones in which the author was not personally involved, have been appropriately investigated, resolved, and the resolution documented in the literature.

## Conflict of Interest

The authors declare that the research was conducted in the absence of any commercial or financial relationships that could be construed as a potential conflict of interest.

## Publisher’s Note

All claims expressed in this article are solely those of the authors and do not necessarily represent those of their affiliated organizations, or those of the publisher, the editors and the reviewers. Any product that may be evaluated in this article, or claim that may be made by its manufacturer, is not guaranteed or endorsed by the publisher.
